# H/D Isotope Effects in Hydrogen Bonded Systems

**DOI:** 10.3390/molecules18044467

**Published:** 2013-04-16

**Authors:** Lucjan Sobczyk, Monika Obrzud, Aleksander Filarowski

**Affiliations:** Faculty of Chemistry, University of Wrocław, Joliot-Curie 14, 50-383 Wrocław, Poland; E-Mails: monika.obrzud@chem.uni.wroc.pl (M.O.); aleksander.filarowski@chem.uni.wroc.pl (A.F.)

**Keywords:** hydrogen bonding, isotope effects, infrared spectra, NQR and NMR spectra, ferroelectric properties

## Abstract

An extremely strong H/D isotope effect observed in hydrogen bonded A-H^…^B systems is connected with a reach diversity of the potential shape for the proton/deuteron motion. It is connected with the anharmonicity of the proton/deuteron vibrations and of the tunneling effect, particularly in cases of short bridges with low barrier for protonic and deuteronic jumping. Six extreme shapes of the proton motion are presented starting from the state without possibility of the proton transfer up to the state with a full ionization. The manifestations of the H/D isotope effect are best reflected in the infra-red absorption spectra. A most characteristic is the run of the relationship between the isotopic ratio ν_H_/ν_D_ and position of the absorption band shown by using the example of NHN hydrogen bonds. One can distinguish a critical range of correlation when the isotopic ratio reaches the value of ca. 1 and then increases up to unusual values higher than 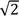. The critical range of the isotope effect is also visible in NQR and NMR spectra. In the critical region one observes a stepwise change of the NQR frequency reaching 1.1 MHz. In the case of NMR, the maximal isotope effect is reflected on the curve presenting the dependence of *Δδ* (^1^H,^2^H) on *δ* (^1^H). This effect corresponds to the range of maximum on the correlation curve between *δ*H and *ΔpK_a_* that is observed in various systems. There is a lack in the literature of quantitative information about the influence of isotopic substitution on the dielectric properties of hydrogen bond except the isotope effect on the ferroelectric phase transition in some hydrogen bonded crystals.

## 1. Introduction

**Figure 1:**
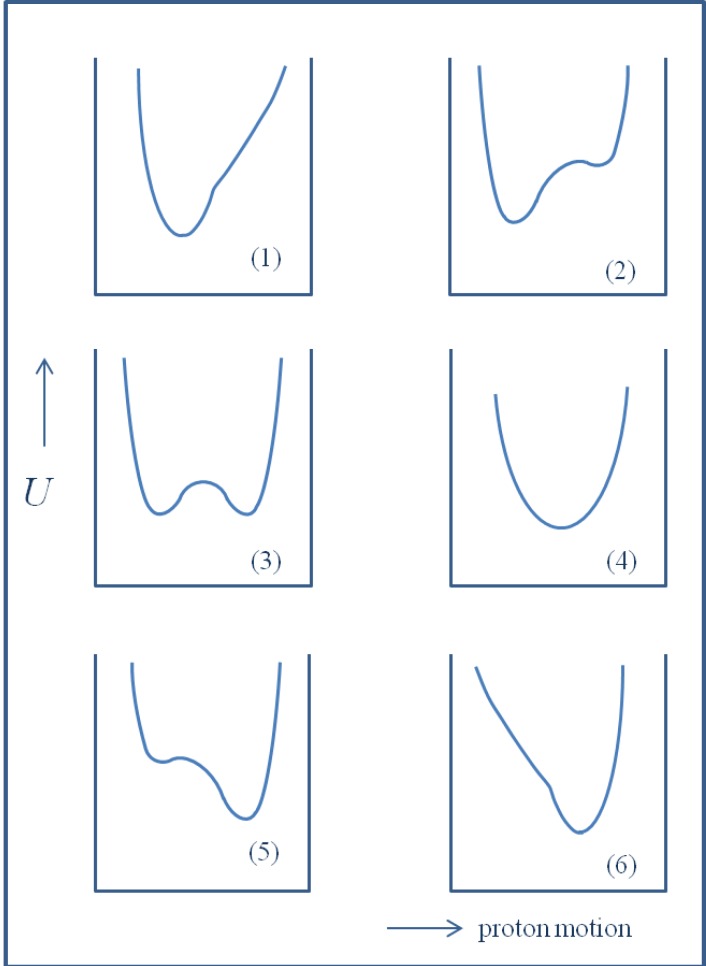
Potential energy (*U*) curves for the proton/deuteron motion depending on the donor-acceptor ability going form weak bridges up to fully ionized state.

There is a rich literature devoted to various aspects of the hydrogen bond effects, including isotope effects. This includes monographs [1,2,3,4,5,6,7,8,9], as well as critical reviews [10,11,12,13,14,15,16,17,18,19,20,21]. The specificity of isotope effects in hydrogen bonded systems comes into prominence particularly due to an anharmonicity of the potential for the hydrogen/deuteron motion as well as due to the tunneling effect. Such a specificity is a result of the bridge atom motion in the A-H^…^B complex with the potential which can have different shapes, as has been shown in Figure 1.

It seems that potential curves 1 and 3–4 possess particularly great importance. In the case of curve number 1 we have to do with a single asymmetric minimum. This is a most popular case of hydrogen bond. The examples 3–4 are related to symmetrical or close to symmetrical hydrogen bonds with two minima and a low barrier (3) or with a single minimum (4). The three other remaining cases are, in some way, a mirror reflection of hydrogen bonds when the hydrogen/deuteron atom is localized closer to the acceptor atom B.

**Figure 2:**
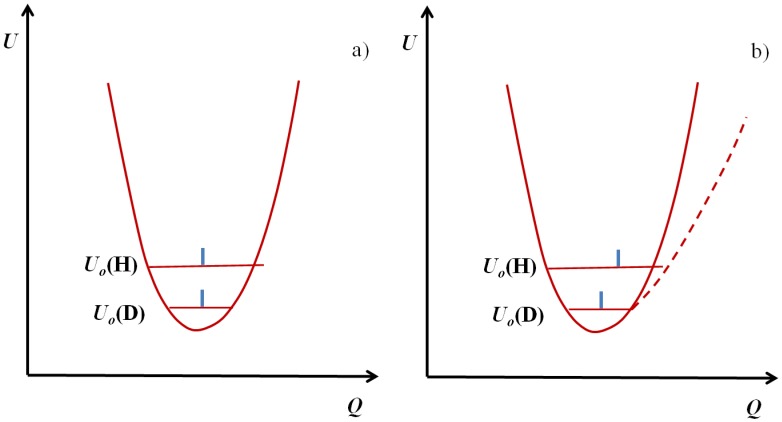
Confrontation of the harmonic (**a**) and anharmonic (**b**) curves with indication of vibrational energy levels and positions occupied by protons and deuterons. *Q* represents the coordinates of H/D atoms.

One should emphasize here that almost as a rule the deuterated bridges are weaker than protonated ones, but this is not a general rule. In the case of harmonic or close to harmonic potential with a single minimum the deuterated bridges are slightly stronger than protonated ones. This is reflected in Figure 2a. Thus the zero point energy level of protonic vibrations is located above the level of deuteronic ones.

**Figure 3:**
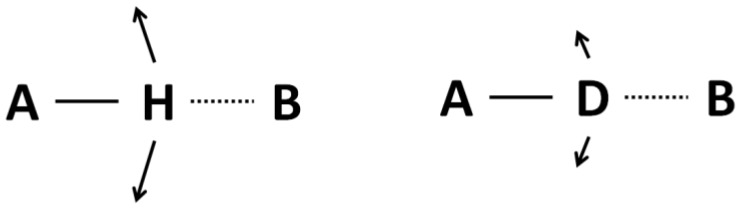
Illustration of the isotope H/D effect on the bending vibrations. The amplitude of A–D vibration is less than that of an A-H one.

For both cases they are located close to the center of the potential curve. The difference in both cases is however substantial enough: namely the amplitude of protonic vibrations is greater than that of deuteronic ones. As a consequence this leads to the situation where a deuterated bridge is a little more stable, but the most important fact is the difference in the amplitude of bending vibrations shown in Figure 3. Undoubtedly the A-D bending vibrations make them a less stable then A-H do. 

**Figure 4:**
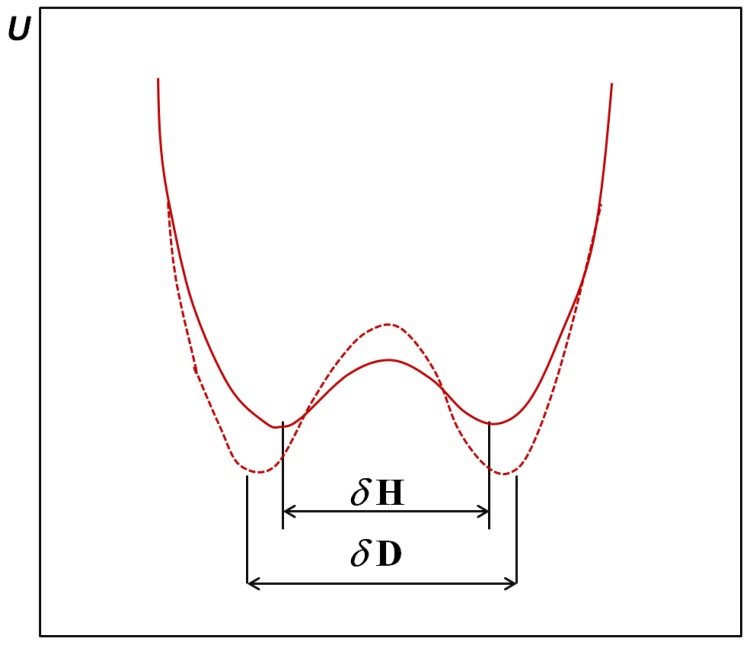
Potential energy curves for strong symmetrical double minimum hydrogen bonds with indicated distances between minima.

In Figure 2b we presented the case of anharmonic vibrations compared with the previous case. On the zero point energy level of vibrations the A atom is shifted to the proton acceptor atom B, which leads to a strengthening of the bridge. In the case of the double minimum potential (curve 3) which takes place in the case of very strong, short bridges, the situation is as shown in Figure 4 according to [22].

The deuteration of the bridge causes on the one hand a separation of the energy minima and on the other hand an increase of the barrier and lowering of the levels of the potential energy minima. These effects are closely connected with the proton/deuteron tunneling ability. When the distance between the A-B bridge atoms is very small, the square of the wave function for the proton can show one maximum and an intense deuteron tunneling. In the case of the shortest bridges both hydrogen and deuterium are characterized by a single maximum of the square of the wave function.

## 2. H/D Isotope Effects in Hydrogen Bonds Reflected in Infrared Absorption Spectra

Most sensitive and differentiated phenomena of the isotope effects in hydrogen bonded systems A-H^…^B are the infra-red absorption spectra. In the case of harmonic vibrations *ν*(AH)/*ν*(AD) values are close to 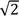. When hydrogen bonds are formed this ratio usually undergoes some reduction. When the hydrogen bond is very strong the value of the isotopic ratio (ISR) reaches a minimum close to unity and then starts to jump intensely.

**Figure 5:**
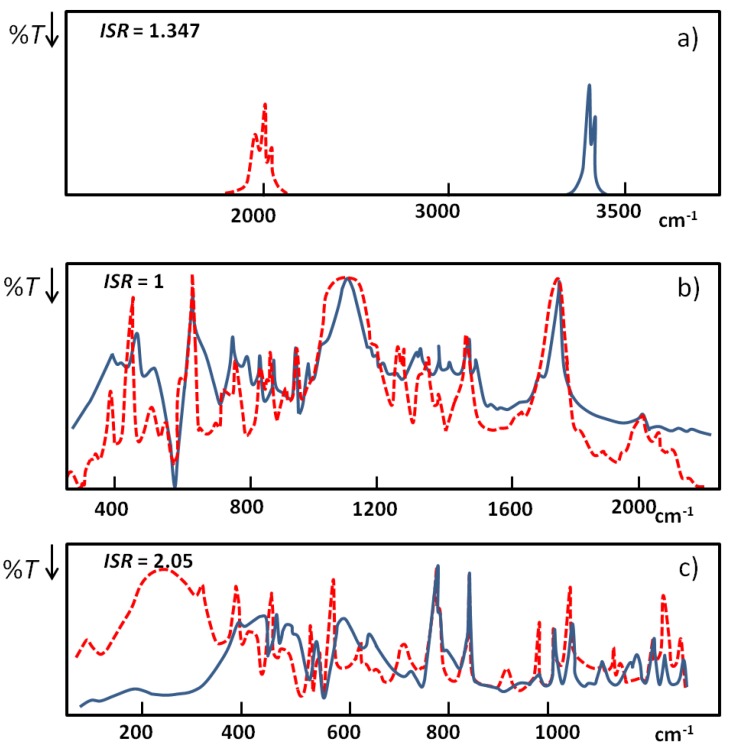
Illustration of the behavior of IR spectra for selected extreme examples of NHN hydrogen bonds: (**a**) associates of diphenylamine; (**b**) quinuclidin-3-one-hemiperchlorate; (**c**) HPF_6_ salt of 1,8-bis(dimethylamino)naphthalene; for NHN blue and for NDN red curves.

In Figure 5 [23] three extreme examples of NHN hydrogen bonded systems characterized by different anharmonicity are presented: (a) associates of diphenylamine, (b) quinuclidin-3-one-hemiperchlorate, (c) HPF_6_ salt of 1,8-bis(dimethylamino)naphthalene; for NHN blue and for NDN red curves. The positions of broad bands are estimated based on their gravity centers.

**Figure 6:**
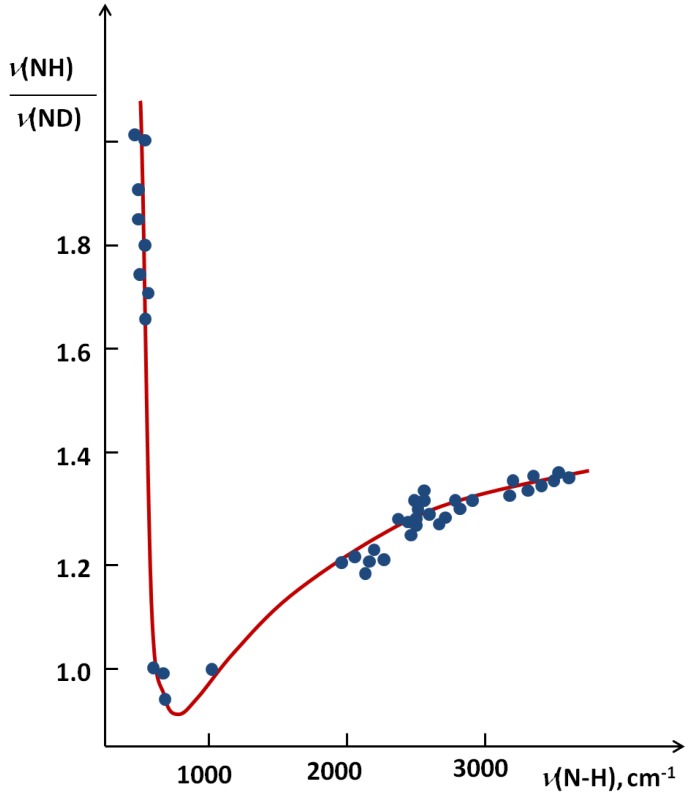
The correlation between the *ISR* value and *ν*(NH) for NHN systems.

In Figure 6 [24] a correlation between the isotopic ratio *ISR ν*(NH)/*ν*(ND) from the literature data for the NHN systems as a function of *ν*(NH) is presented.

**Figure 7:**
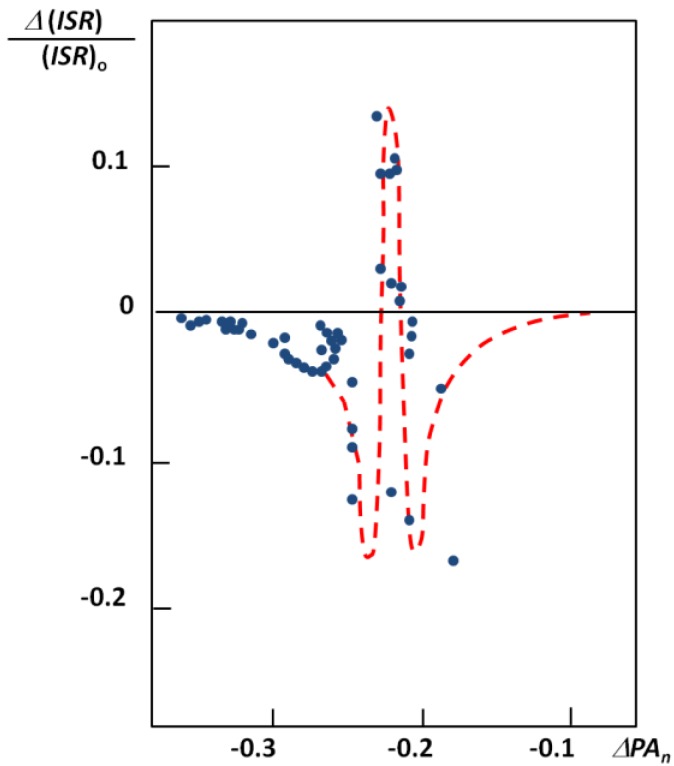
Correlation between the relative isotopic ratio and the Pimentel parameter for X-H^…^B complexes in argon matrices.

The figure seems to be a perfect example how the *ISR* value changes with an increase of the hydrogen bond strength. The lowest *ISR* values (below unity) are related to the bridges with double energy minimum and very low barrier for the proton transfer. Without doubts the isotopic ratio is an important influence in short bridges that possess tunneling effects. Interesting results of studies on XH^…^B complexes (where B means F, Cl, Br, I) performed in low temperature matrices are connected with this problem. The results are used is searches for correlations between the relative change of *ISR* defined as (*ISR_c_-ISR_o_*)/*ISR_o_* (*ISR_c_* and *ISR_o_* mean isotopic ratios for complexes and free molecules) and normalized proton affinity (PA) defined by Pimentel as [25]:

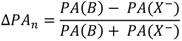

where *PA*(B) and *PA*(X^−^) represent the proton affinity of a base B and anion X^−^. The correlation between the relative isotopic ratio and normalized Pimentel parameter is presented in Figure 7 [25]. As shown the positive extreme values of 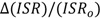 correspond to a defined critical region which is related for systems with *ISR* > 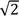.

Information about isotopic effects affecting other parameters for hydrogen bonded systems can be found in the review [12].

## 3. H/D Isotope Effects in NQR and NMR Spectra

**Figure 8:**
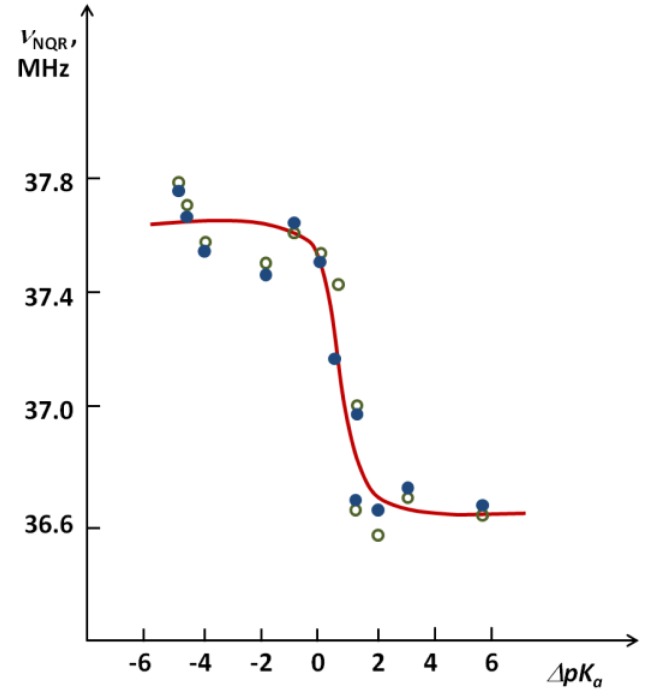
The average value of NQR frequencies for ^35^Cl nuclei depending on *ΔpK_a_* in the case of pentachlorophenol complexes with a number of various proton acceptors.

One clearly observes the isotope effect in the nuclear quadrupole resonance (NQR) spectra as well as in nuclear magnetic resonance (NMR) spectra, although not so intensively as in the infra-red spectra. In the case of NQR spectra we decided to select as an example the dependence of the NQR frequency on the value of 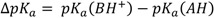 as shown in Figure 8 [26]. A critical *ΔpK_a_* region where a stepwise decrease of the *ν_NQR_* value takes place is visible.

In Figure 8 points for deuterated systems in relation to points corresponding to nondeuterated ones are presented. The differences are not giant but very well reflected. It is characteristic that in agreement with the expectation, the differences of NQR frequencies after exceeding the critical region are opposite compared to the subcritical region with higher *ν_NQR_* values. The highest difference of *ν_NQR_* appeared for one complex from the critical region (ca 0.3 MHz). We would like to highlight that the stepwise change of NQR frequency in the critical region reaches as much as 1.1 MHz. The curve reminds one, according to the expectation, of the dependence of the dipole moment increase on *ΔpK_a_*.

**Figure 9:**
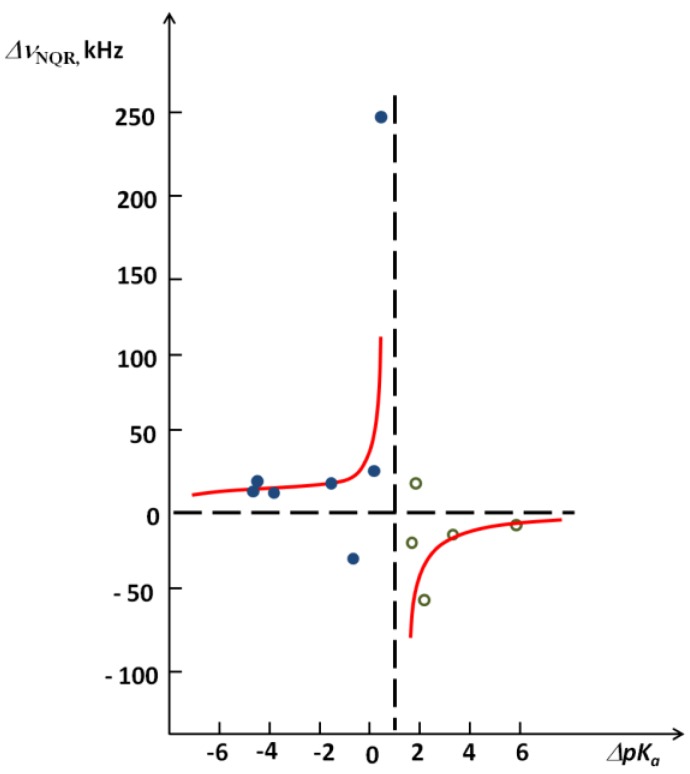
Diagram of the dependence of ^35^Cl NQR frequency after deuteration on *ΔpK_a_* for hydrogen bonded complexes of pentachlorophenol.

The critical region of the dependence of Δ*ν(NQR)* on *ΔpK_a_* after deuteration for complexes of pentachlorophenol can also be presented clearly in Figure 9 [26].

**Figure 10:**
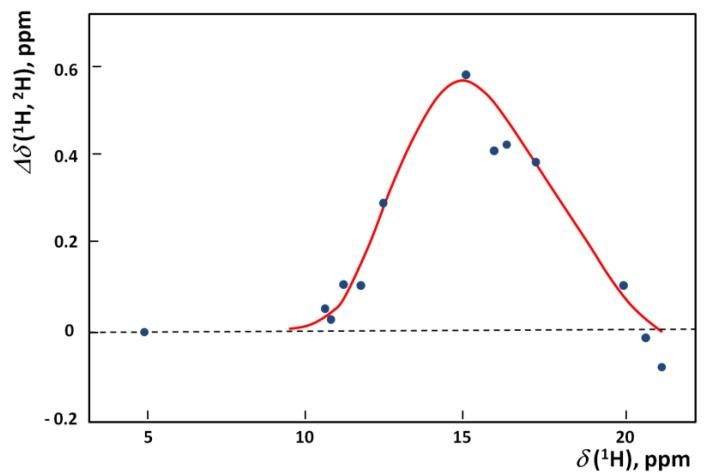
The dependence of *Δδ*(^1^H,^2^H) on *δ*(^1^H) for hydrogen bonded systems.

The influence of isotopic substitution is very well visible in NMR (both primary as well as secondary effects). From among various quantitative analyzes we selected the example of correlation between *Δδ*(^1^H,^2^H) and *δ*(^1^H) for systems with OHO hydrogen bonds. Such a correlation is presented in Figure 10 [27].

The maximal isotope effect in the diagram takes place in the region of the maximum on the correlation curve between *δ*H and *ΔpK_a_* that is observed in various systems. The high values of primary isotopic effect as in Figure 10 are interpreted in terms of the double minimum potential with low barrier and as a consequence of the tunneling effect.

## 4. Ferroelectric Properties of Hydrogen Bonded Crystals

Spectacular isotope effects appear in ferroelectric crystals with hydrogen bonds. The phase transition temperature ferroelectric-paraelectric phases is most frequently connected with ordering of protons. In the paraelectric phase we have to do with a symmetric potential curve that implies a disorder of protons, while in ferro- or antiferroelectric phases this curve becomes asymmetric: protons are localized at particular bridge atoms (usually oxygen atoms). Deuteration leads as a rule to an increase of critical temperature [28,29]. In the case of OHO bridges maximal effects one observes are in the range of 2.5–2.6 Å length. In the case of very short hydrogen bonds we do not observe in general the ordering of protons, even at the lowest temperatures. These spectacular isotope effects correlate very well with neutron diffraction studies which show that the positions of potential minima after deuteration are more separated. The interpretation of above phenomena was elaborated in [22] based on the tunneling effect. 

## 5. Conclusion

A variety of potential energy curves along the A-H^…^B bridges does exist depending on the proton donor-acceptor ability of A and B atoms (Figure 1). The shapes of the potential curves express the fact that the hydrogen bonds are extremely sensitive to the electron density distribution within whole molecules and to the environment. Among various systems particularly interesting seem to be the hydrogen bonds with a double or single central minimum potential. These systems can be treated as critical ones because they show unusual properties and difference between protonated and deuterated species.

Most characteristic is the H/D isotope effect in the infra-red absorption spectra. In the present review we concentrated our attention among others on the NHN bridges showing the correlation between the isotopic ratio ISR *ν(NH)/ν(ND)* and the *ν(NH)* value (Figure 6). This Figure is best illustrative when analyzing the H/D isotope effect. The minimum on the curve (close to unity) can be treated as a critical point. Critical points are also seen in correlation in Figure 7, Figure 8, Figure 9, Figure 10 showing dependences of NQR and NMR data on the proton donor-acceptor ability parameters. 
